# The complete mitochondrial genome of *Sarcophaga tuberosa* (Diptera: Sarcophagidae)

**DOI:** 10.1080/23802359.2019.1644218

**Published:** 2019-07-25

**Authors:** Xie Kai, Wang Shiwen, Yanjie Shang, Lipin Ren, Yadong Guo

**Affiliations:** a Xiangya School of Medicine, Central South University, Changsha, China;; b Department of Forensic Science, School of Basic Medical Sciences, Xinjiang Medical University, Ürümqi, China;; c Department of Forensic Science, School of Basic Medical Sciences, Central South University, Changsha, China

**Keywords:** Mitochondrial genome, *Sarcophaga tuberosa*, Sarcophagidae

## Abstract

*Sarcophaga tuberosa* has certain sanitary and epidemiological significance and takes proteins from decaying matter. In this study, we first sequenced and analyzed the complete mitochondrial genome (mitogenome) of *S. tuberosa*. The length of mitogenome was 15,173 bp, consisting of A (39.5%), G (9.4%), T (37.0%), and C (14.1%), and the order and orientation of genes were identical with that from the mittogenomes of flesh flies previously reported. Phylogenetic analyses showed that *S. tuberosa* was clustered separately, but which was closed to the species of *Sarcophaga portschinskyi*. This study provided better support for further understanding of phylogenetic relationships and enriched the mitogenome database of flesh flies.

*Sarcophaga (Liosarcophaga) tuberosa* Pandelle 1896 belongs to the genus *Sarcophaga*, which is a parasitic natural enemy insect that controls the development of pine moth, with entomological and horticultural importance (Sierpiñska 1998). The mitigenome has been used in an effort to enhance accessibility to method of species identification, comparing to traditional morphological approaches (Alessandrini et al. 2008; Ren et al. 2018). In this study, the complete mitogenome of *S. tuberosa* was 15,173 bp in length (Genbank No. MK820723), encompassing 37 genes (13PCGs, two rRNA genes, 22 tRNA genes, and an A + T-rich region). In addition, it displayed characteristics of A (39.5%), G (9.4%), T (37.0%), and C (14.1%). We supplemented the data sets of flesh flies and provided further insights on phylogeny and taxonomy of Sarcophagidae.

The adult of *S. tuberosa* specimens was trapped in Beijing, China (39°26′N; 115°25′E). Each specimen was identified according to traditional morphological analysis by an expert. DNA extraction was performed using the QIANamp Micro DNA Kit, and the complete mitogenome was conducted on an Illumina HiSeq 2500 Platform (Ren et al. 2019). All the voucher specimens were deposited in the Guo’s lab (Changsha, Hunan, China), with a unique code (CSU19040905).

Phylogenetic analyses of *S. tuberosa* and 11 Sarcophagidae species were performed based on 13PCGs using Neighbor-joining (NJ) inference method, with *Calliphora vomitoria* and *Chrysomya pinguis* as the outgroup (Figure 1). Phylogenetic analyses showed that *S. tuberosa* was clustered separately, but which was closed to the species of *Sarcophaga portschinskyi* (Buenaventura et al. 2016). This study provided better support for further analyzing the molecular phylogenetic relationships and enriched data sets of flesh flies.

**Figure 1.**
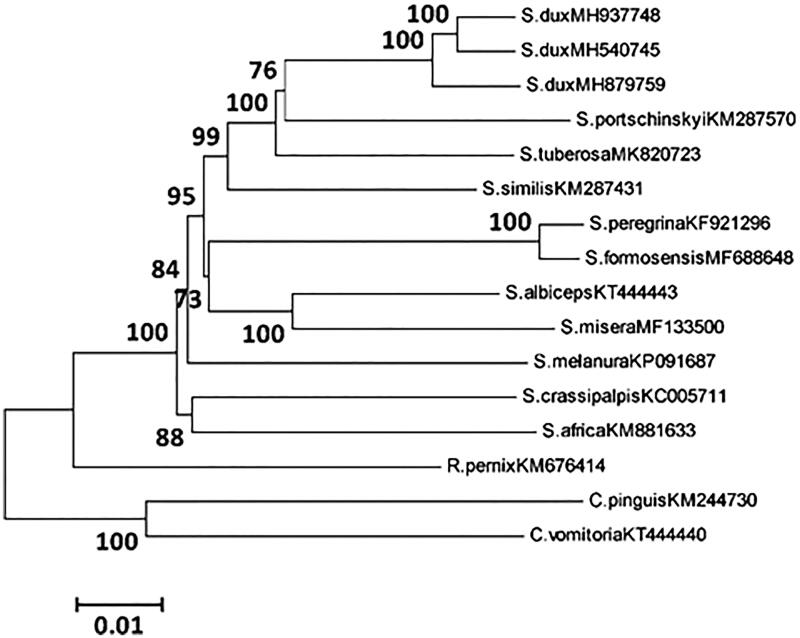
Phylogenetic analyses were constructed using NJ method based on 13PCGs, with two blowflies as the outgroup.
